# Management of Febrile Neutropenia in a Tropical Country

**DOI:** 10.1016/j.htct.2024.11.118

**Published:** 2024-12-19

**Authors:** Eduardo Magalhães Rego

**Affiliations:** a Instituto do Câncer do Estado de São Paulo (ICESP), São Paulo, SP, Brazil; b Instituto D´Or de Pesquisa e Ensino (IDOR), São Paulo, SP, Brazil; c Laboratório de Investigações Médicas 31, Faculdade de Medicina da Universidade de São Paulo (LIM 31 FM USP), São Paulo, SP, Brazil

In the present issue of HTCT, we publish the recommendations from the Infectious Diseases Committee of the Brazilian Society of Hematology, Blood Transfusion and Cell Therapy (ABHH) for managing febrile neutropenia in hematologic patients ^1^. The frequency and impact of infectious complications on the outcome of the treatment of hematological malignancies in the context of constrained resources have been reported in several retrospective studies 2, 3, 4. In addition, in several comparative studies, infectious complications were identified as leading to shorter overall survival in real-world studies in Latin America.^5^^,^^6^

The Consensus was based on the Delphi methodology, which is a systematic process of forecasting using the collective opinion of panel members. ABHH recommendations cover the initial workup, prophylaxis, empiric antibiotic, and antifungal therapy, modifications in the empiric regimen, and criteria for discontinuing antimicrobial therapy. Although guidelines for managing febrile neutropenia are not new, it is important to point out that the ABHH Committee was careful to include variables relevant to the Brazilian context that may be of value for other tropical and subtropical regions. Recently, Hamburger et al. ^7^ conducted a systematic review of publications addressing Candida species distribution and antifungal susceptibility among Brazilian patients with candidemia. The authors emphasized that access to diagnostic tests was limited, and only one-third of the studies on candidemia could report antifungal susceptibility testing results. In vitro, resistance to echinocandins was reported in 1.5% of the isolates, whereas fluconazole resistance rates ranged from 0 to 43%. Bergamasco et al.^8^ reported 94 cases of invasive fungal disease (IFD) among 664 hematologic patients and 316 hematopoietic stem cell transplant (HSCT) recipients. The frequency of IFD among patients with allogeneic HSCT, autologous HSCT, acute leukemia and other hematologic malignancies was 8.9%, 1.6%, 17.3%, and 6.4%, respectively. Aspergillosis was the leading IFD (53.2%), followed by fusariosis (18.1%), candidiasis (10.6%), and cryptococcosis (8.5%). The ABHH recommendations include the use of posaconazole as the anti-mold agent of choice in patients receiving intensive chemotherapy for induction remission in acute myeloid leukemia/myelodisplastic syndrome (AML/MDS), fluconazole for antifungal prophylaxis after autologous HSCT, and voriconazole as the anti-mold agent of choice after allogeneic HSCT. Moreover, the ABHH panel agreed that serial (2-3 times weekly) serum galactomannan antigen tests should be performed in neutropenic patients at high risk of developing invasive aspergillosis who are not receiving antifungal prophylaxis against Aspergillus species. In contrast, serial serum galactomannan should not be part of the routine in neutropenic patients receiving a mold-active azole such as voriconazole, posaconazole or isavuconazole because of the risk of false-positive results.

The article is especially important for hematologists in training, as it provides a comprehensive framework for managing a critical condition in hematologic patients. By integrating the latest evidence, this consensus document will undoubtedly serve as a valuable resource for improving the care of patients with hematologic malignancies, ensuring more effective, context-aware interventions.

## References

[1] Nucci M, Arrais-Rodrigues C, Bergamasco MD, Garnica M, Glória ABF, Guarana M (2024). Management of Febrile Neutropenia: Consensus of the Brazilian Society of Hematology, Blood Transfusion and Cell Therapy – ABHH. Hematol Transfus Cell Ther.

[2] Mendes FR, da Silva WF, da Costa, Bandeira de Melo R, Silveira DRA, Velloso EDRP, Rocha V (2022). Predictive factors associated with induction-related death in acute myeloid leukemia in a resource-constrained setting. Ann Hematol.

[3] Rabagliati R, Salazar G, Pérez-Lazo G, Iturrieta MP, Portillo D, Soria-Segarra C (2024). An Emergent Change in Epidemiologic and Microbiological Characteristics of Bloodstream Infections in Adults With Febrile Neutropenia Resulting From Chemotherapy for Acute Leukemia and Lymphoma at Reference Centers in Chile, Ecuador, and Peru. Open Forum Infect Dis.

[4] Garnica M, da Cunha MO, Portugal R, Maiolino A, Colombo AL, Nucci M. (2015). Risk factors for invasive fusariosis in patients with acute myeloid leukemia and in hematopoietic cell transplant recipients. Clin Infect Dis.

[5] Aristizabal P, Fuller S, Rivera R, Beyda D, Ribeiro RC, Roberts W. (2015). Improving Pediatric Cancer Care Disparities Across the United States-Mexico Border: Lessons Learned from a Transcultural Partnership between San Diego and Tijuana. Front Public Health.

[6] Sanz MA, Montesinos P, Kim HT, Ruiz-Argüelles GJ, Undurraga MS, Uriarte MR (2015). All-trans retinoic acid with daunorubicin or idarubicin for risk-adapted treatment of acute promyelocytic leukaemia: a matched-pair analysis of the PETHEMA LPA-2005 and IC-APL studies. Ann Hematol.

[7] Hamburger FG, Gales AC, Colombo AL. (2024). Systematic Review of Candidemia in Brazil: Unlocking Historical Trends and Challenges in Conducting Surveys in Middle-Income Countries. Mycopathologia.

[8] Bergamasco MD, Pereira CAP, Arrais-Rodrigues C, Ferreira DB, Baiocchi O, Kerbauy F (2021). Epidemiology of Invasive Fungal Diseases in Patients with Hematologic Malignancies and Hematopoietic Cell Transplantation Recipients Managed with an Antifungal Diagnostic Driven Approach. J Fungi (Basel).

